# Prioritization of Vaccines for Inclusion into China’s Expanded Program on Immunization: Evidence from Experts’ Knowledge and Opinions

**DOI:** 10.3390/vaccines10071010

**Published:** 2022-06-24

**Authors:** Chao Ma, Junhong Li, Nan Wang, Yamin Wang, Yudan Song, Xiang Zeng, Canjun Zheng, Zhijie An, Lance Rodewald, Zundong Yin

**Affiliations:** 1 National Immunization Program, Chinese Center for Disease Control and Prevention, Beijing 100050, China; machao@chinacdc.cn (C.M.); lijh@chinacdc.cn (J.L.); wangym2@chinacdc.cn (Y.W.); songyd@chinacdc.cn (Y.S.); anzj@chinacdc.cn (Z.A.); rodewaldl@chinacdc.cn (L.R.); 2 Shanghai Municipal Center for Disease Control and Prevention, Shanghai 201204, China; wangnan@scdc.sh.cn; 3 Zhuhai Center for Disease Control and Prevention, Zhuhai 519000, China; zengx022@163.com; 4 Chinese Field Epidemiology Training Program (CFETP), Chinese Center for Disease Control and Prevention, Beijing 100050, China; 5 Division of Infectious Disease, Chinese Center for Disease Control and Prevention, Beijing 100050, China; zhengcj@chinacdc.cn

**Keywords:** vaccine, inclusion, Expanded Program on Immunization, Delphi, indicator system

## Abstract

Background: Vaccine developers in China have made an increasing number of infectious diseases preventable through vaccination. An appropriate decision-making procedure is necessary for making wise decisions on whether to introduce new vaccines into the Expanded Program on Immunization (EPI). When there are several vaccines that could potentially be considered, a scientifically justifiable mechanism is needed for prioritizing and sequencing vaccines for consideration. Methods: We used a modified Delphi technique (MDT) to develop and refine an indicator system to prioritize vaccines and make policy recommendations concerning their introduction into China’s EPI system. From January through May 2021, thirty-nine experts were recruited and participated in a two-round Delphi survey that was based on a set of candidate indicators obtained through a literature review and reference to the WHO vaccine introduction recommendations. Using the resulting indicator system, we conducted a third consultation with a multi-disciplinary group of experts who scored five program-eligible candidate vaccines to determine prioritization and sequencing for consideration of inclusion into the EPI. Results: Response rates of the thirty-nine experts were 100% and 97.4% across the two rounds. Authority coefficients from rounds one to three were over 0.70, reflecting the high accuracy and reliability of the consultation. Coordination coefficients of importance scores for primary, secondary, and tertiary indicators were 0.486, 0.356, 0.275 in round one, and 0.405, 0.340, and 0.236 in round two. According to the scores from 30 experts using our indicator system, the sequence and scores (1–10 scale, 10 highest) of 5 candidate vaccines were varicella (6.91), meningococcal conjugate AC (6.83), Hib (6.74), influenza (6.56), and EV71 (6.17) vaccines. Conclusions: A modified Delphi technique effectively built a scientific, rational, comprehensive, and systematic indicator system for prioritizing vaccine candidates for consideration of inclusion into the EPI. The rank order will be used by the technical working groups of China’s National Immunization Advisory Committee to sequentially develop and present Evidence-to-Recommendation tables for making policy recommendations.

## 1. Introduction

China launched the National Immunization Program (NIP)—also called the Expanded Program on Immunization (EPI)—in 1978. The program initially protected children against six vaccine-preventable diseases (VPDs) with four vaccines: bacille Calmette–Guerin vaccine (BCG), oral poliovirus vaccine (tOPV), diphtheria–tetanus–pertussis vaccine (DTP), and measles vaccine (MV). In 2002, protection was extended against an additional VPD by including the hepatitis B vaccine (HepB), and in 2007, against five more VPDs by including hepatitis A (HepA), meningococcal polysaccharide (MPV), Japanese encephalitis (JEV), and rubella and mumps vaccines [1]. The EPI is guided by the National Health Commission (NHC), which has a responsibility to make evidence-based decisions regarding further expansion of the EPI, the introduction of non-EPI vaccines into the EPI system, and the replacement of current EPI vaccines with new ones.

In China, the government has a duty to provide safe and effective EPI vaccines at no cost to families. Parents and legal guardians have a corresponding duty to vaccinate their children with EPI or equivalent non-EPI vaccines. Non-EPI vaccines are paid for by families, and families are under no obligation to use non-EPI vaccines [2]. Coverage levels of EPI vaccines are consistently and uniformly high [3], but coverage of non-EPI vaccines is lower and variable, resulting in less effective disease prevention. For example, one dose of mumps vaccine was included in the EPI, but some provinces included two doses, resulting in superior protection [4]; varicella vaccine is not included in the EPI, but some provinces provide one or two doses, resulting in lower incidence rates [5]; and during the 6 years prior to the 2016 introduction of inactivated poliovirus vaccine into the EPI, 157 children were paralyzed by tOPV-caused vaccine-associated paralytic polio [6].

Over the years, vaccine developers have made an increasing number of infectious diseases preventable through vaccination, with more than 30 non-EPI vaccines currently market authorized in China. However, new vaccine introduction has lagged the availability of new vaccines, missing important opportunities for preventing serious VPDs. There are at least eight vaccines that could be considered for introduction into the current set of EPI vaccines: Hemophilus influenzae type b (Hib), inactivated influenza vaccine (IIV), varicella vaccine (VarV), meningococcal polysaccharide conjugate (MPCV), enterovirus type 71 (EV71), pneumococcal conjugate vaccine (PCV), human papillomavirus vaccine (HPV), and rotavirus vaccine (Rota). Decisions for vaccine introduction must be science-based. Using evidence for immunization decision-making procedures and national policy development is a global health priority. National Immunization Technical Advisory Groups (NITAGs) are independent technical advisory bodies for this purpose [7]. The National Immunization Advisory Committee (NIAC), China’s NITAG, was established in 2017 to adjust the vaccines included in the EPI [8]. To this end, appropriate decision-making procedures must be used to make wise decisions on vaccines to be included in the EPI.

The World Health Organization (WHO) developed guidance for making decisions and plans to introduce a vaccine into a national immunization program [9]. However, there is no tool for prioritizing and sequencing vaccines for introduction when there are several vaccines eligible for consideration. Expert consultation has been used to make strategic work plans for NITAGs. The Delphi method is a systematic and qualitative way to elicit opinions from a group of experts through several rounds of questions. It is applicable to public health problems that cannot be solved by commonly used analytic methods [10], and it is widely used in the construction of evaluation indicator systems [11,12,13].

As a deliberative committee, NIAC cannot consider the inclusion of all potentially program-eligible vaccines simultaneously—vaccines will be sequentially considered and introduced if favorably recommended. To develop a rational medium-term policy work plan for NIAC, we used a modified Delphi technique (MDT) to systematically obtain and synthesize the opinions of experts to prioritize and sequence potentially program-eligible vaccines for consideration of EPI introduction. We report results from the MDT study and discuss the next steps for the evidence-based introduction of new vaccines into China’s EPI system.

## 2. Methods

From January through May 2021, we used a modified Delphi technique to refine a set of quantitative indicators for prioritizing vaccines to be considered for inclusion in China’s EPI system. Indicators are specific lines of evidence useful for making vaccine policies. Examples include the burden of disease preventable by the vaccine, the economic value of the vaccine, the safety of the vaccine, and the willingness of parents to vaccinate their children with the vaccine. Through a literature review, discussion with EPI experts, and reference to the WHO principles and considerations for adding a vaccine to a national immunization program [9], we assembled a list of 36 candidate indicators for considering the inclusion of a vaccine into China’s EPI system (Supplementary Table S1). From January through April 2021, we conducted two rounds of surveys with a panel of experts to refine the set of indicators. Subsequently, thirty experts were asked to score EPI-eligible vaccines using the refined indicator set.

### 2.1. Selection of Delphi Experts

We used a purposive sampling technique [10] to select knowledgeable individuals to serve on a panel of experts. Selected experts were researchers from disease prevention and control institutions with the academic rank of associate senior or above, with at least a bachelor or higher degree, who had more than 10 years of experience in vaccination practices and immunization research, and who were willing to participate continuously throughout the consultation. Ultimately, 39 experts in public health, immunology, epidemiology and health statistics, health economics, clinical medicine, and infectious diseases were recruited to participate in the Delphi process.

### 2.2. Delphi Survey to Establish Indicator System

The purpose of the Delphi survey was to develop and refine an indicator system for prioritizing and sequencing vaccines for introduction into the EPI system.

#### 2.2.1. Questionnaire Development

We categorized candidate indicators into primary, secondary, and tertiary indicators, with lower-level indicators being more fine-grained than upper-level ones. The five top-level (primary) indicators were characteristics of the disease prevented by the vaccine, characteristics of the vaccine, ability of the vaccination to be implemented in the EPI system, international experience with the vaccine, and potential societal impact of the vaccine (Supplementary Table S1).

The Delphi questionnaire consisted of four parts: (1) instructions for the Delphi survey to introduce the study domains and describe how to complete the survey; (2) the questionnaire, with candidate indicators for evaluating the inclusion of a vaccine into China’s EPI system; (3) questions about experts’ authority in the EPI, including familiarity with indicators and judgment criteria for the indicator; and (4) additional question about the experts, such as age, professional title, position, education, major, and duration of work in their professional field.

#### 2.2.2. Expert Consultations

We conducted both rounds of expert consultation using either electronic questionnaires or face-to-face interviews. In the first round, experts scored the indicators on their assessments of importance, familiarity, and judgment of appropriateness. We utilized a five-point Likert scale [12,14] for the importance of each indicator and calculated means, covariances, and full-mark ratios (proportion of responses giving “full marks”, i.e., maximum scores in the Likert scale) for each candidate indicator. We used a 5-point score (1.0, 0.8, 0.6, 0.4, 0.2), ranging from extremely familiar to unfamiliar, to indicate experts’ familiarity. For experts’ judgment basis and degree of influence, we used three-point scores that weighted practical experience (0.5, 0.4, or 0.3) higher than theoretical knowledge (0.3, 0.2, or 0.1), which was weighted higher than referring to domestic and international literature (0.1, 0.1, or 0.1) and “instinctual feeling” (0.1, 0.1, or 0.1) [15,16].

In round one, an indicator was retained if it met at least one of three threshold criteria, which were based on means, covariances, and full-mark ratios of the importance scores for candidate indicators. Round one questionnaires were analyzed to achieve a consensus, and new candidate indicators suggested by the experts during round one were included in round two. The round one consensus was based solely on importance.

In round two, experts assigned weights to the candidate indicators according to their familiarity with the prioritization study and their sources of knowledge about the indicators (i.e., direct experience with the VPD, theoretical knowledge, opinions of others from the literature, or their own feeling or opinion).

### 2.3. Prioritization of Candidate Vaccines

With the indicator system established and refined, we conducted a third expert consultation to determine the prioritization of candidate vaccines for NIAC’s consideration for policy recommendation of inclusion into the EPI system.

#### 2.3.1. Candidate Vaccines

We used 3 criteria to screen 41 non-EPI vaccines for inclusion in the third round of expert consultations: (1) whether the potential candidate vaccine is a childhood vaccine, as we are only considering childhood vaccines for inclusion into China’s EPI system; (2) whether the vaccine is produced by at least three domestic manufacturers; and (3) whether the vaccine is recommended by the WHO for inclusion into every national immunization program or for widespread use in countries with epidemiological situations similar to those in China. Five vaccines met all three criteria: VarV, MPCV-AC, Hib, IIV, and EV71 vaccines.

#### 2.3.2. Expert Consultation

We selected at random 30 of the 39 experts who participated in rounds one and two to participate in round three. The experts assigned scores from one to ten to the tertiary indicators; scores were multiplied by weighting coefficients and summed across the indicators to yield a single numeric value for each candidate vaccine. Relative values of the scores were used to develop a sequence for NIAC deliberation on policy recommendations.

### 2.4. Data Analysis

The intent of the analyses was to use experts’ knowledge and self-confidence in their knowledge and refine the three-level set of indicators so that the indicators can be used in a quantitative survey of experts that will yield a prioritization and sequencing of vaccines for consideration of their inclusion in the EPI.

Data entry and statistical analyses were performed using Excel 2016 and IBM SPSS 22.0. We calculated frequencies, rates, means, full mark ratios, standard deviations, and coefficients of variation for the importance scores of indicators at each level.

The reliability and representativeness of the expert consultations were measured as follows: The positivity coefficient (response rate), calculated as the ratio of the number of questionnaires delivered to the number of those returned, was used to appraise experts’ enthusiasm. The authority coefficient (Cr), calculated as Cr = (Cs + Ca)/2, where Ca and Cs are arithmetical means of experts’ familiarity scores and their sources of knowledge scores, was used to assess experts’ perceived degree of authority [17]. Expert consultation results were considered reliable when Cr ≥ 0.70 [18]. Consensus across expert panel participants was evaluated using Kendall’s coefficient of concordance (W), which evaluates the degree of consistency among the experts and consistency of scoring results during a Delphi process [19]. Kendall’s W ranges from 0 (no agreement) to 1 (complete agreement) [19,20,21]; non-parametric testing (chi-square) was used to determine whether the resulting W values were due to chance. Arithmetic means, coefficients of variation, and full-mark ratios (the proportion of experts giving the maximum score of an item) of the importance scores reflect the degree of concentration of expert opinions on each indicator. An indicator was considered highly acceptable when the standard deviation of the indicator’s importance score was <1, and the coefficient of variation was <0.2 [22]. For all analyses, *p* < 0.05 was considered statistically significant. The hybrid weighting coefficients of each indicator were calculated using the product method, in which indicator coefficients at the three levels were serially multiplied to yield a hybrid weighting.

### 2.5. Ethical Considerations

The study was approved by the Ethics Committee of China CDC (ERB number 2020). Experts provided written informed consent for participation in the study. Data were anonymized prior to analysis.

## 3. Results

### 3.1. Demographic Characteristics of the Experts

Table 1 shows the characteristics of the 39 round one and two senior experts. The experts included people in international organizations; national-, provincial-, and municipal-level CDCs; health administrative departments; and scientific research institutions. In total, 33 (84.61%) experts had master’s degrees or above, and 23 (58.98%) had more than 20 years of working experience in their field. The 30 (76.92%) experts who were selected at random from the round one/two expert panel to participate in the round three Delphi survey were similar to the original 39 experts.

### 3.2. Reliability and Representation of Expert Consultation

Table 2 shows the positivity coefficients, authority coefficients, importance scores of indicators, coefficients of variation, and concordance coefficients (W) of consultation for rounds one, two, and three. These measures assess the reliability and representation of expert consultation. All authority coefficients from rounds one through three were over 0.70, reflecting high accuracy and reliability of consultation. In round one, standard deviations of the importance scores were >1 for 35% of the indicators, and coefficients of variation were >0.2 for 50% of the indicators. In round two, standard deviations of all indicators became <1, and coefficients of variation for only four indicators were >0.2. Thus, the indicators became highly acceptable. The Kendall coordination coefficients (W) evaluated for all three levels of indicators in the first two consultation rounds and for the five candidate vaccines in the third consultation round showed that the experts’ scoring levels were highly similar (all *p* < 0.001).

### 3.3. Indicator System Construction

Table 3 shows the boundary values for the importance of the candidate indicators. After the first Delphi survey round, four candidate indicators were removed because they were not within the boundary values—one primary-level indicator, two secondary-level indicators, and one tertiary-level indicator. Through expert consultation and face-to-face interviews, six more tertiary-level indicators were removed, three pairs of tertiary indicators were merged, and three secondary-level and two tertiary-level indicators were added. After the second Delphi survey, two indicators were removed that were not within the boundary values—one primary level and one secondary level indicator. After two rounds of expert consultation, the final indicator system consisted of 3 primary-, 13 secondary-, and 26 tertiary-level indicators with weighting coefficients (Table 4).

### 3.4. Prioritization of Vaccines

The thirty questionnaires received in round three all met validity criteria. The order and score of the five candidate vaccines were VarV (6.91), MPCV-AC (6.83), Hib (6.74), IIV (6.56), EV71 (6.17), in which the possible range of scores was from one to nine. The resulting scores were in a narrow range of values that indicated all five vaccines were considered favorable by the thirty experts. Table 4 shows the final set of indicators at the primary, secondary, and tertiary levels with the means of the experts’ indicator values shown for each of the five candidate vaccines.

## 4. Discussion

Our study used a modified Delphi technique and developed a survey-based system for achieving a common expert perspective that can be used to prioritize vaccines for introduction into China’s EPI system. Factors most influential to the experts were similar to the WHO guidelines for new vaccine introduction [9]: features of the disease (epidemiological characteristics, burden of disease, and importance for prevention and control); features of the vaccine (vaccine characteristics, performance, availability, cost-effectiveness, and international and provincial experience); and features of the EPI program itself (financing, cold chain capacity, and public confidence). Concordance among the experts was high and increased with successive rounds of consultation. Using the resulting indicator system, a panel of thirty experts prioritized varicella, meningococcal conjugate AC, Hib, influenza, and EV71 vaccines for rank-order consideration of policy recommendations for inclusion into China’s EPI system.

### 4.1. Study in Context

Expert consultations have been used to make strategic work plans for NITAGs. For example, in 2005, US CDC’s Advisory Committee on Immunization Practices (ACIP) held an independent consultation for expanding influenza vaccine recommendations to additional target populations that would eventually lead to universal influenza vaccine recommendations [23]. The consultation helped focus several years of effort by the ACIP influenza working group.

There are technical aids for decision-making procedures regarding immunization programs. One of the best known is PROVAC, from the Pan American Health Organization (PAHO). PROVAC was developed in 2004 and incorporates a health and economic model for determining whether to include a vaccine into an EPI system [24]. A major innovation of PROVAC is the use of economic reasoning for decision making [25]. In contrast, the modified Delphi technique we used is a more general means to achieve a quantitative decision. Candidate factors for decision making were prespecified in our MDT based on the performed literature review and consultation with experts, but their weightings were dynamic and allowed for the reflection of experts’ self-assessments of knowledge, strengths, and limitations.

The US Institute of Medicine (now the National Academy of Medicine) developed a quantitative, multi-attribute ranking tool (SMART) in 2012 to support decisions on vaccine development. The tool was an adjunct for expert decision making to quantify the potential values of a vaccine yet to be developed [26]. Similar to our use of MDT to prioritize the ordering of introducing vaccines into the EPI, SMART prioritized vaccine development. While SMART is a tool for upstream decision making, our MDT is for downstream decision making—only vaccines that are “ready” to be included in China’s EPI were considered in our study. Vaccines we considered had to be licensed, be in use as a non-program vaccine, and have at least three domestic vaccine manufacturers—a reduced scope that streamlines the problem being solved by MDT. For example, the domestic production capacity and security of supply of pneumococcal conjugate, HPV, and rotavirus vaccines are currently insufficient for consideration of their inclusion into China’s EPI system. Therefore, these vaccines were not included in our MDT study.

Robust frameworks exist for determining whether or not to include a vaccine in a national EPI system. The most mature and frequently emulated framework is the WHO Evidence to Recommendation (E2R) Framework [27]. Individual vaccine decision making is a critically important problem; the use of the E2R framework requires a substantial amount of resource-intensive scientific work. Our MDT study was not intended to replace the E2R framework. Rather, it was intended to focus the scientific support work for NIAC by concentrating on vaccines that are most “ready” and most necessary for China’s EPI system. Completing E2R tables for these five vaccines will be the next work of NIAC’s secretariat. The usefulness of our MDT study is that the upcoming E2R work can be placed into a multi-year work plan.

### 4.2. Strengths and Limitations

A strength of our study is the quantification of expert opinions using a known methodology, and the use of quantitative indicators on evidence that will be needed by NIAC for consideration of vaccine inclusion. Measures of concordance and consistency were used to understand the degree of consensus among the experts, adding confidence to the validity of the results. Weightings of the indicators were adjusted based on experts’ self-assessments of their own knowledge, strengths, and weaknesses, in effect, adjusting weightings to the expert’s self-confidence in their knowledge, and thus reducing guesswork. A limitation is that only one panel of experts was used for this consultation. A different panel with differing areas of expertise could yield different prioritizations of the vaccines. However, the MDT does not influence NIAC policy work—only the sequencing of vaccines for consideration of policy recommendations.

### 4.3. Program Implications and Next Steps

Although vaccines will be recommended by NIAC for inclusion in the EPI based on their own evidence and merit, prioritization from our study provides a set of five vaccines that are “ready” to have their evidence summarized in an E2R table and considered by NIAC for recommendation into China’s EPI. Being “ready” implies that there is production capacity for national introduction and that experts consider these vaccines to be beneficial to children and meritorious for the EPI. Our study results will inform the overall work plan for NIAC technical working groups, focusing the work to expedite policy recommendations on the inclusion of vaccines into the EPI.

Our study identified scientific areas in which the evidence base is insufficient. Cost-effectiveness data for including vaccines into the EPI are insufficient for some of the prioritized vaccines, and improvement in disease burden surveillance for bacteria disease (Hib) is needed. Partnership with academic institutions and the development of “in-house” expertise will be necessary to ensure NIAC has the evidence needed for policy making. For vaccines that are included in the EPI system, additional research is warranted on vaccine product selection strategies that can help ensure a healthy market environment for the program and manufacturers.

In conclusion, using a modified Delphi technique, 39 experts refined a set of indicators that enabled the National Immunization Program to obtain an expert prioritization for sequencing vaccines into the EPI system in China. In rank order of priority, from the experts’ points of view, varicella, meningococcal conjugate AC, Hib, influenza, and EV-71 vaccines should be considered by NIAC working groups to make policy recommendations for inclusion in the EPI for routine vaccination of children. The modified Delphi technique may also be applicable for the best use of multiple COVID-19 vaccines in the National Immunization Program.

### 4.4. Highlights

New vaccine introduction into China’s EPI system has lagged the availability of new vaccines, resulting in missed opportunities for preventing serious VPDs. A modified Delphi technique was used to redefine a set of quantitative indicators for prioritizing vaccines to be considered for inclusion in China’s EPI system. Using the resulting Delphi indicators, experts rank-ordered varicella, meningococcal conjugate AC, Hib, influenza, and EV71 vaccines for consideration of introduction into the EPI. The results will inform the overall work plan for NIAC technical working groups, focusing the work to expedite policy recommendations on the inclusion of prioritized vaccines into the EPI.

## Figures and Tables

**Table 1:** Demographic characteristics of the experts.

Characteristic	Number (%)	
	Rounds 1 and 2	Round 3
Gender		
Male	20 (51.28)	15 (50.00)
Female	19 (48.72)	15 (50.00)
Highest educational degree		
Doctor	18 (46.15)	14 (46.67)
Master	15 (38.47)	11 (36.67)
Undergraduate	6 (15.38)	5 (16.67)
Work experience (years)		
10–20	16 (41.02)	12 (40.00)
21–30	14 (35.90)	9 (30.00)
31–40	9 (23.08)	9 (30.00)
Title ^a^		
Senior title	34 (87.18)	26 (86.67)
Deputy senior title	5 (12.82)	4 (13.33)
Research field		
Epidemiology and health statistics	11 (28.21)	1 (3.33)
Public health	10 (25.64)	11 (36.67)
Immunization program	6 (15.38)	10 (33.33)
Health economics	5 (12.82)	3 (10.00)
Clinical medicine	4 (10.26)	3 (10.00)
Infectious diseases	3 (7.69)	2 (6.67)

^a^ Senior title included chief physician, researcher, and professor. Deputy senior titles include deputy chief physician, deputy researcher, and associate professor.

**Table 2:** Reliability and representation of expert consultation.

Factors	Round 1			Round 2			Round 3		
	Value	χ^2^	*p*	Value	χ^2^	*p*	Value	χ^2^	*p*
Positivity coefficients	100%	-	-	97.44%	-	-	100%	-	-
Authority coefficients	0.84	-	-	0.86	-	-	0.85	-	-
Importance scores of indicators	3.44–4.95	-	-	3.49–4.83	-	-	-	-	-
Standard deviation	0.22–1.25	-	-	0.33–0.98	-	-	-	-	-
Coefficients of variation	0.05–0.36	-	-	0.07–0.28	-	-	-	-	-
Concordance coefficients (W)									
Primary indicators	0.486	75.852	<0.001	0.405	30.758	<0.001	-	-	-
Secondary indicators	0.356	138.659	<0.001	0.340	155.242	<0.001	-	-	-
Tertiary indicators	0.275	374.802	<0.001	0.236	224.091	<0.001	-	-	-
Hib	-	-	-	-	-	-	0.388	291.047	<0.001
IIV	-	-	-	-	-	-	0.303	227.128	<0.001
VarV	-	-	-	-	-	-	0.301	225.475	<0.001
EV71	-	-	-	-	-	-	0.253	189.857	<0.001
MPCV-AC	-	-	-	-	-	-	0.221	166.017	<0.001

**Table 3:** Boundary values for the importance of all-level indicators.

Factor	Round 1			Round 2		
	Primary	Secondary	Tertiary	Primary	Secondary	Tertiary
Mean	3.85–4.95	3.56–4.95	3.44–4.87	4.11–4.72	3.49–4.82	3.82–4.83
Threshold value	3.95	3.82	3.76	4.17	3.73	4.01
Coefficient of variation	0.05–0.25	0.05–0.29	0.07–0.36	0.09–0.17	0.07–0.28	0.07–0.24
Threshold value	0.24	0.26	0.29	0.16	0.23	0.19
Full mark ratio (%)	28.21–94.87	20.51–92.31	12.82–84.62	10.53–52.63	5.26–68.42	2.63–78.95
Threshold value	30.50	26.72	25.54	13.90	7.01	13.70
Standard deviation	0.22–0.96	0.22–1.03	0.34–1.25	0.41–0.69	0.33–0.98	0.36–0.94

**Table 4:** Indicator system and Delphi results for vaccine inclusion into EPI.

Level of Indicator			Interpretation of the Tertiary Indicator	Combined Weight	Candidate Vaccine				
Primary	Secondary	Tertiary			Hib	MPCV-AC	IIV	VarV	EV71
Diseases prevented by the vaccine			-	0.420					
	Epidemiological characteristics		-	0.126					
		Endemic area	Larger endemic area → higher possibility of including the vaccine.	0.028	7.17	5.97	8.38	8.37	6.73
		Morbidity rate	Higher morbidity → higher possibility of considering the vaccine.	0.029	6.00	5.20	7.63	7.70	6.30
		Population mortality rate	Higher mortality → higher possibility of considering the vaccine.	0.037	6.00	7.33	5.07	4.43	5.47
		Case disability rate	Higher disability rate → higher possibility of considering the vaccine.	0.032	5.73	7.38	4.37	4.20	4.90
	Economic burden			0.120					
		Direct economic burden	Higher direct economic burden → higher possibility of considering the vaccine.	0.069	6.43	6.70	6.30	6.07	5.87
		Indirect economic burden	Higher indirect economic burden → higher possibility of considering the vaccine.	0.051	6.47	6.53	6.07	6.10	5.70
	Public health priority			0.115					
		In national public health list	Whether control and prevention of the disease are included in China’s national public health priority list. If yes, the disease has a higher possibility of being considered.	0.062	6.63	6.68	7.22	7.43	6.30
		Public health emergency event	Whether the disease can cause public health emergency events. If yes, the disease has a higher possibility of being considered.	0.053	5.07	6.27	7.57	7.57	6.73
	Non-vaccine interventions (NVI)			0.059					
		Cost of NVI	Higher costs of NVI (for example, hand washing, face mask, medicines) → higher possibility of being considered.	0.020	5.53	5.77	5.90	5.97	5.67
		Effectiveness of NVI	Less effective NVI → higher possibility of being considered.	0.020	5.80	5.90	5.73	6.07	5.67
		Sustainability	Less sustainable of implementing NVI → higher possibility of being considered.	0.019	5.57	5.60	5.93	6.20	5.70
Candidate vaccine				0.371					
	Vaccine performance and characteristics			0.118					
		Efficacy and Effectiveness	More effective → higher possibility of considering the vaccine.	0.044	7.80	7.90	6.33	8.00	7.07
		Persistence	Longer vaccine protection persistence → higher possibility of considering the vaccine.	0.031	7.43	7.40	4.60	7.47	6.40
		Safety	Safer → higher possibility of considering the vaccine.	0.044	8.50	8.13	8.23	8.10	8.23
	Cost-effectiveness			0.083					
		Vaccination cost	Lower cost → higher possibility of considering the vaccine.	0.037	6.47	6.43	6.90	6.40	6.17
		Cost-effectiveness	More cost-effective → higher possibility of considering the vaccine.	0.046	7.40	6.92	6.67	7.47	6.53
	Availability of vaccine supply			0.081					
		Maximum supply	Vaccine supply meets NIP need → higher possibility of being considered.	0.040	7.77	7.40	7.10	7.53	7.10
		Sustainability	Supply sustainable → higher possibility of being considered.	0.040	7.63	7.47	7.20	7.50	7.33
	International experience			0.042					
		WHO recommends	Recommended by WHO → higher possibility of being considered.	0.024	8.93	7.77	7.27	7.57	4.40
		Most countries include the vaccine in NIP	Other countries have introduced the vaccine → higher possibility of being considered.	0.018	8.67	7.13	6.80	6.90	3.73
	Domestic experience			0.046					
		Provincial inclusion in local immunization program	Introduced into local immunization program by some provinces → higher possibility of being considered.	0.046	5.076	6.23	5.77	6.83	4.33
Vaccination implementation				0.209					
	Financial issues			0.081					
		Financial affordability	Operational costs are affordable → higher possibility of being considered.	0.081	6.47	6.57	6.10	6.53	5.97
	Acceptability			0.047					
		Willingness for vaccination	Public willing to receive the vaccination → higher possibility of being considered.	0.047	7.23	7.33	6.80	7.70	6.63
	Ethical consideration			0.038					
		Benefit–risk ratio	Benefit far exceeds risk → higher possibility of being considered.	0.038	7.70	7.38	7.17	7.53	6.43
	Capability of implementation			0.043					
		Cold chain administration	Cold chain affordable → higher possibility of being considered.	0.020	5.90	6.20	6.07	6.17	6.10
		Immunization service system	Immunization services (e.g., human resources, information system, and surveillance) are affordable → higher possibility of being considered.	0.023	6.80	6.70	6.37	6.77	6.67
Total scores for each vaccine					6.74	6.83	5.56	6.91	6.17

## Data Availability

Not applicable.

## Supplementary Materials

The following supporting information can be downloaded at: https://www.mdpi.com/article/10.3390/vaccines10071010/s1, Table S1: Candidate indicators for considering inclusion into the EPI before the first round of expert consultation.
