# Supplementary material for: Prioritization of Vaccines for Inclusion into China’s Expanded Program on Immunization: Evidence from Experts’ Knowledge and Opinions
Source: Vaccines (Basel). 2022 Jun 24;10(7):1010. doi: 10.3390/vaccines10071010 (PMC9318118; doi:10.3390/vaccines10071010)
Supplement: Supplementary file 1 [file vaccines-10-01010-s001.zip › vaccines-1779683-supplementary.pdf]

Supplementary file

**Table S1. Candidate indicators for considering inclusion into the EPI before the first round of expert consultation.**

| Level of indicator | | | Interpretation of the tertiary indicator |
|---|---|---|---|
| **Primary** | **Secondary** | **Tertiary** | |
| **Characteristics of disease** | | | - |
| | Importance of prevention and control | | - |
| | | Population affected | Higher proportion of children cases → higher possibility of being considered |
| | | Endemic area | Larger endemic area → higher possibility of being considered |
| | | Morbidity rate | Higher morbidity → higher possibility of being considered |
| | | Population mortality rate | Higher mortality → higher possibility of being considered |
| | | Case fatality rate | Higher fatality rate → higher possibility of being considered |
| | | Case disability rate | Higher disability rate → higher possibility of being considered |
| | | Direct economic burden | Higher direct economic burden → higher possibility of being considered |
| | | Indirect economic burden | Higher indirect economic burden →higher possibility of being considered |
| | | Listed in national public health priority | Control and prevention of the disease is listed in China's national public health priority list → higher possibility of being considered |
| | | Public health emergency event | The disease cause public health emergency events often → higher possibility of being considered |
| | Non-vaccine interventions (NVI) | | |
| | | Cost of NVI | Higher costs of NVI (for example, hand washing, face mask, medicines) → higher possibility of being considered |
| | | Effectiveness of NVI | Less effective NVI → higher possibility of being considered |
| | | Persistence of NVI effectiveness | Longer persistence of NVI effectiveness → higher possibility of being considered |
| | | Sustainability | Less sustainable of implementing NVI → higher possibility of being considered |
| | | Adverse influence of NVI | Lower adverse influence of NVI → higher possibility of being considered |
| | | Efficiency of NVI | More efficient of NVI generating benefits → higher possibility of being considered |
| **Characteristics of vaccine** | | | |
| | Vaccine performance and characteristics | | |
| | | Efficacy and Effectiveness | More effective → higher possibility of being considered |
| | | Persistence | Longer vaccine protection persistence → higher possibility of being considered |
| | | Safety | Safer → higher possibility of being considered]\ |
| | | Type | Combined vaccine rather than single antigen → higher possibility of being considered |
| | Vaccine use | | |
| | | Target population | Wider target age range → higher possibility of being considered |
| | | Vaccination cost | Lower cost → higher possibility of being considered |
| | | Cost-effectiveness | More cost-effective → higher possibility of being considered |
| **Supporting conditions** | | | |
| | Availability of vaccine supply | | |
| | | Production capacity | Domestic vaccine production capacity meets NIP need → higher possibility of being considered |
| | | Sustainability | Supply sustainable → higher possibility of being considered |

| Level of indicator | | | Interpretation of the tertiary indicator |
|---|---|---|---|
| **Primary** | **Secondary** | **Tertiary** | |
| | Financial issues | | |
| | | Financial affordability | Operational costs affordable → higher possibility of being considered |
| | | Sustainability | Finance sustainable → higher possibility of being considered |
| | Human resource and infrastructure | | |
| | | Technical capability | Lower technical requirement for vaccination staff → higher possibility of being considered |
| | | Cold chain system | Lower requirement for cold chain → higher possibility of being considered |
| | | Surveillance system | With existing surveillance system for the disease, vaccination coverage, and AEFI → higher possibility of being considered. |
| **International experience** | | | |
| | WHO recommends | | |
| | | Recommending inclusion into NIP | Recommended including into NIP by WHO → higher possibility of being considered |
| | | Universal vaccination | Recommended vaccinating universally by WHO → higher possibility of being considered |
| | Experience of other countries | | |
| | | Proportion of countries having included into NIP | Higher proportion→ higher possibility of being considered |
| **Social influence** | | | |
| | Acceptability | | |
| | | Public awareness | Higher awareness rate about the vaccine and disease → higher possibility of being considered |
| | | Willingness for vaccination | Public willing to receive the vaccination → higher possibility of being considered |
| | Ethical consideration | | |
| | | Benefit risk ratio | Benefit far exceeds risk → higher possibility of being considered |
